# Two Hours of Separation Prior to Milking: Is This Strategy Stressful for Jennies and Their Foals?

**DOI:** 10.3390/ani11010178

**Published:** 2021-01-14

**Authors:** Sharacely de Souza Farias, Ana Carolina Dierings Montechese, Thiago Bernardino, Paulo Henrique Mazza Rodrigues, Chiara Albano de Araujo Oliveira, Adroaldo José Zanella

**Affiliations:** 1 Department of Preventive Veterinary Medicine and Animal Health, School of Veterinary Medicine and Animal Science, Campus “Fernando Costa”, University of São Paulo, Pirassununga 13635-900, Brazil; ana.montechese@usp.br (A.C.D.M.); thiagobernardino@usp.br (T.B.); adroaldo.zanella@usp.br (A.J.Z.); 2 Department of Animal Nutrition and Production, School of Veterinary Medicine and Animal Science, Campus “Fernando Costa”, University of São Paulo, Pirassununga 13635-900, Brazil; pmazza@usp.br; 3 Department of Preventive Veterinary Medicine and Animal Production, School of Veterinary Medicine and Animal Science, Federal University of Bahia, Salvador 40170-110, Brazil; oliveirachiara@gmail.com

**Keywords:** animal welfare, behaviour, donkey, cortisol, milking management

## Abstract

**Simple Summary:**

The economic importance of donkeys has decreased in Brazil, which has led to their mass abandonment. Asinine milk production is a potential solution to the reintroduction of donkeys into the Brazilian social and economic scenario. The milk has nutraceutical properties that make it valuable for human consumption, and thus a donkey dairy industry is likely to help stop their abandonment. That said, in any such industry, the welfare of jennies maintained for milk production must be guaranteed. Few studies have been published measuring the impact of milking management on the welfare of jennies and foals, and the potential behavioural and physiological challenges it may cause. It is also unknown whether these animals adapt to the milking routine. The goal of this study was to assess the impact of separating Pêga jennies from their foals for 2 h on indicators of welfare. Animal welfare was analysed through behavioural and hormonal assessments, their potential adaptive responses and effects on milk yield. Few significant alterations were found in behaviour, salivary cortisol concentrations, or milk yield as a result of the 2-h separation, which could indicate that the welfare of the animals was not compromised; however, the adaptation of jennies and foals to separation stress remains to be fully verified. The 2-h separation period, based on the reported data, is possibly not a stressful experience for the assessed group of Pêga jennies and foals. The reported protocol, which included frequent positive interactions with the animals, may be useful to assure acceptable animal welfare levels for donkeys in small-scale dairy production settings.

**Abstract:**

The goal of this study was to assess whether or not a separation period of 2 h is stressful for jennies and foals, as measured by changes in behaviour, salivary cortisol, and milk production. This study was reviewed and approved by the Committee for the Use and Care of Animals in Research (CEUA) of the School of Veterinary Medicine and Animal Science of the University of São Paulo. Fourteen multiparous Pêga jennies (245 kg average body weight) and their foals were assessed from day 45 to 135 of lactation. Dams and foals were separated for 2 h prior to milking. Behavioural assessments and saliva samples were collected before and after separation, every 15 days, resulting in 14 samples per individual animal. Behavioural states (affiliative and inactivity) and events (agonistic, abnormal, eliminative and vocalisations) of the jennies were observed during 6 min in both periods. Moreover, milk yield was measured. Few significant behavioural and salivary cortisol changes were observed, and milk yield was not affected by cortisol levels in response to the separation. The 2-h separation period, on the basis of the collected variables, did not appear to be stressful for the assessed group of Pêga jennies or foals; however, their ability to adapt to milking routine stress remains to be investigated.

## 1. Introduction

Donkeys have been losing their relevance in Brazilian social and economic scenarios, having been less used in recent decades in their traditional role as animals of draft and burden. This trend can be ascribed, essentially, to the diffusion of mechanisation in agriculture [1], and the subsequent decrease in the number of donkeys used on rural properties. As a result, they have been omitted from official data and statistics collected by the Brazilian government [2], and their abandonment has been intensified, leading to increasing numbers of wandering animals with compromised welfare [3].

There are three registered Brazilians donkey breeds: Nordestino, Paulista, and Pêga [4]. Out of these breeds, the Pêga has been most developed for its genetic potential. It was developed in Brazil in 1810 [5], and is composed of medium sized donkeys, primarily bred to supply the market with mules [6]. Pêga donkeys are most commonly found in the Southeast region, and although they have a clear economic importance, productive donkey farms in Brazil are scarce and these animals are still generally left out of the social and economic scenario in the country.

In other countries, such as France and Italy, donkeys are still valued because of their milk which is used for human nutrition [7,8]. Asinine milk possesses similar chemical and organoleptic qualities to human milk [7,9], representing one of the best nourishment options, besides maternal milk, for human babies [10] that cannot be breast-fed and for consumers suffering from cow milk protein allergies [11,12]. The milk of Brazilian Pêga donkeys may have similar nutritional potential to that of Italian and French breeds, and thus their use for sustainable, high welfare, donkey dairy production is a possible means of reintroducing donkeys as an important species in the Brazilian socioeconomic scenario. The use of animals for milk production must guarantee their welfare. There is a limited number of studies regarding stressors that trigger physiological and behavioural changes, generated by the milking management in donkeys [13].

The milk storage capacity in this species is low (less than 2.5 L) [14], thus milk production is dependent on its removal from the mammary gland, generally by milking or suckling. In the latter, milk ejection is triggered by a foal’s sucking, which triggers the release of oxytocin that in turn induces the contraction of myoepithelial cells [15]. Milking of jennies by humans, in terms of both human and animal safety and for optimal milk extraction, is more manageable when foals are not physically present [16].

In order to achieve efficient milking, jennies must be milked after 2 to 3 h of physical separation from their foals [17]. Long intervals between milking events may cause a rise in intra-udder pressure, inducing early cessation of glandular activity [18], due to the udder size and its low storage capacity. Therefore, donkeys may need to be milked multiple times a day [19].

The social structure of donkeys is composed of a territorial-based system [20,21,22], with complex hierarchies within groups [23]. The only permanent bond among donkey social structures is between jennies and their foals [24].

In precocious animals such as donkeys, the neonatal period is characterized by intense interactions between mothers and newborns, which are important for bonding and allow for the development of autonomy in the offspring, including motor, sensorial and cognitive processes [24]. In natural conditions, jennies begin approaching their foals less frequently after the first day post-partum, grazing at further distances while the foals rest and allowing them to interact with other animals [25]. During the first five days of life, donkey foals suckle every 3 to 10 min, and every 20 to 30 min by the 10th day [26]. In mule foals, the suckling frequency between 4 and 17 weeks varies between two and three bouts per hour [27].

Behavioural and social impairments have been reported in ungulates separated from their mothers for 2.5 h after birth [28,29]. The separation between jennies and foals may be stressful [30,31], and these animals could respond via behavioural and physiological changes [32]. When the restoration of homeostasis in response to a stressor is difficult, such as when animals cannot move to a more favourable environment, they may express behavioural signals such as vocalisations [33], stereotypic behaviour [34], increased inactivity [35,36,37] and altered social interactions [38]. Behavioural responses facilitate physiological adaptations, which may manifest via the activation of the autonomous nervous system and neuroendocrine system [39]. The stimulation of central circuits involving the amygdala, hypothalamus and periaqueductal gray (PAC) result in an increased frequency of eliminative behaviour [40], and increased releases of corticotropin-releasing factor (CRF), from the hypothalamus, and adrenocorticotropic hormone (ACTH), from the pituitary gland culminate in an increase in the secretion of glucocorticoids, such as cortisol [41], from the adrenal glands. Alveolar milk ejection could also be altered by fear or stress, due to the influence of these endocrine factors on oxytocin concentration and myoepithelial contraction [42,43].

To our knowledge, there are no scientific studies concerning the adaptive responses of jennies and foals to repeated separation and milking procedures, and so it is crucial to determine their behavioural and physiological changes when exposed to this routine. Therefore, further investigations assessing the welfare effects, if any, of milking procedures on dairy jennies and their foals are required.

This study aimed to investigate whether a separation period of 2 h in a manual milking system is stressful for Pêga jennies and foals, i.e., whether it generated changes in behaviour, or caused changes in salivary cortisol concentration and milk production, and thus, if the use of donkeys for sustainable, high welfare, donkey dairy production is a model for their economic reintroduction in Brazil. For this purpose, a manual milking protocol was proposed and implemented.

## 2. Animals, Materials and Methods

This study was reviewed and approved by the Committee for the Use and Care of Animals in Research (CEUA) of the School of Veterinary Medicine and Animal Science of the University of São Paulo, under the protocol number CEUA 8696141117 (ID 007216).

### 2.1. Animals, Housing and Management

The study was conducted in Criatório Ximbó, a donkey farm in the city of Laranjal Paulista in the state of São Paulo, Brazil. The city of Laranjal Paulista is located at an altitude of 536 m, at the coordinates 23°02′59″ latitude South and 47°50′12″ longitude West. The local climate is humid subtropical (Köeppen–Geiger classification), the yearly average temperature ranges from 13 ± 4.9 °C to 31 ± 4.7 °C, and the yearly average pluviosity is around 1177 mm.

On the farm, donkeys are kept in a semi-intensive system, and receive nutrition composed of native foliage and *Brachiaria decumbens,* as well as alfalfa hay supplementation.

The donkeys on the farm are kept in pastures during the day and are moved to stalls of 24 m^2^ stalls (6 m × 4 m) overnight, in stable groups of 3 (Stall 1, 8 m^2^ allowance per jenny) to 4 jennies (Stall 2, 6 m^2^ allowance per jenny) and their foals in each stall. In the morning, they are released by simply opening the stall door. All animals assessed in this study were habituated to this daily routine.

The farm had no dairy production activity, and the 60 Pêga jennies on the farm were used for reproduction. Fourteen multiparous Pêga jennies (245 kg average body weight) were studied from day 45 to 135 of lactation. Of the fourteen jennies assessed, seven foaled in February 2018 and were assessed until June, and seven foaled in May and were assessed until September of the same year. No milk was collected during the first month of each foal’s life as it was used exclusively for their nutrition in the month of June, around day 135 of lactation for the first group and day 45 of lactation for the second group, all fourteen jennies and their foals were assessed at once, resulting in some animals being separated for up to 3 h.

For data collection, the established groups of jennies were maintained and minimal changes to the already established farm routine were made.

### 2.2. Experimental Design

On each data collection day, while still inside of the pens, together with their group, the behaviour of jennies in Stall 1 was assessed and saliva was sampled from both jennies and foals. The animals were then separated for 2 h. The same procedures were repeated in Stall 2. In order to separate them, two people stood at the stall door and allowed the jennies to pass through while impeding the foals from following. The jennies were stimulated to leave the stall using visual and sound cues, such as raising hands and clapping, and the foals were stopped by standing in their path. During the separation period, the jennies were loose on the farm, while the foals were kept inside of the group stalls with no visual contact with their dams. Information regarding the stall of each animal, as well as the exact time of release from the stalls, was recorded on each data collection day in order to keep the groups and separation times constant throughout the study.

After 2 h of separation, the jennies were led, one by one, to the milking parlour. This was the first instance they were restrained, utilising a halter and loose lead rope to keep them from leaving the parlour. At this time, their respective foals were brought to the milking parlour from the stalls, in less than one minute, marking the end of the separation period. The jennies’ behaviour was then assessed, and saliva samples were taken from both jennies and foals. The foals were not restrained, but were stopped from suckling by placing a hand between their mouth and the dam’s teat.

It was observed that, from the third collection day onwards, for both the February and May groups, the jennies tended to wait at the milking parlour by the end of the separation time, and did not need to be brought back from elsewhere on the farm.

During milking, an additional safety measure was taken by firmly tying a lead rope to their hind limb and securing it to a fence. The milk yield was noted.

Separation of jennies and foals took place at 10:00 am. Saliva samplings were conducted, before separation, between 8:50 am and 9:50 am, and after separation, between 12:00 pm and 2:00 pm.

Behavioural assessments and saliva samplings were performed from day 45 to 135 of lactation, totalling 14 assessments per animal. All data were collected every 15 days, to assess the possible adaptation of these animals to the stress generated by the milking management routine.

### 2.3. Behaviour Assessments

For the behavioural assessments, jennies were identified with ribbons of different colours attached to their necks. The protocol used for behaviour assessment was focal sampling with continuous recording, performed directly by two trained assessors utilising a check sheet.

The occurrence of behavioural states (long-duration behaviours such as prolonged activities, measured in time intervals between the beginning and end of each episode) and events (instantaneous or short-duration behaviours) were observed in the jennies in the pre- and post-separation periods and were later evaluated.

The observed behavioural states were affiliative behaviour and inactivity, and the events were agonistic, abnormal and eliminative behaviours, as well as vocalisations. These behavioural categories were chosen as they could be affected by the presence of a stressor [33,34,35,36,37,38], and various aspects of behaviour were assessed in order to paint a complete picture of any alterations the jennies exhibited between the pre- and post-separation assessments. It was expected that, if these animals were stressed by the separation, the duration of these behavioural states would be altered with potential increases in inactivity [35,36,37] and decreases in affiliative behaviour [38] post-separation, and the frequency of occurrence of these behavioural events would increase post-separation [33,34]. For these assessments, recording sheets based on an experimental ethogram, developed in this study, were used (Table 1). All observations yielded focal observation data from each animal, with a 6-min duration for each jenny in each assessment [44].

Behaviours considered abnormal were biting the stalls or structures, false licking and pawing. Eliminative actions were urinating and defecating [45].

The recorded vocalisations included various types of vocal communication sounds, such as whinnies, snores, snorts, groans and screams [33].

Social interactions were divided between affiliative and agonistic according to the performed action and response of the receiving animal. In the absence of signs of aggression [46], interactions were considered affiliative, and behaviours linked to aggression were considered agonistic. The observed affiliative interactions were grooming, licking, sniffing, approaching and touching [46,47]. Agonistic interactions were kicking, pushing, chasing, biting and fighting [36]. All social interactions were performed between jennies and foals or other jennies.

After the behavioural assessment of the jennies, saliva was sampled from all animals in the pre- and post-separation periods.

### 2.4. Saliva Sampling

Saliva samples were collected from each animal using an individual sampler developed for this study, which did not require the animals to be restrained. For the jennies, the collector was made of ground *rapadura* (sugarcane candy) wrapped in gauzes and a cotton string. The inclusion of *rapadura* was necessary to stimulate saliva production in the jennies. For the foals, only gauzes and cotton strings were used.

The samplers were presented to all animals by holding them stretched, and both jennies and foals voluntarily approached the assessors to chew on the samplers. Sometimes, the samplers were secured to the animals’ necks with the cotton strings, while the procedure was carried out on the rest of the animals.

Jennies and foals chewed the collectors for 2 min, after which the strings were cut and discarded, and the gauzes were placed in 15 mL Falcon tubes with their respective identifications. The tubes were stored in sealed styrofoam boxes lined with reusable gel ice packs.

### 2.5. Milking

Milking procedures began with the cleaning of the jennies’ udders and teats with soap and water and drying with paper towels. They were milked manually.

The milking stopped once the udders were fully emptied, after which they were cleaned and dried again. After these procedures, each teat was submerged in a post-dipping solution (Dermasoft 2.5%, composed of Povidone-iodine (2.5 g) and purified water (100 mL)) for at least 15 s.

Milk yield was noted for each jenny on every assessment day.

### 2.6. Salivary Cortisol Analysis

The 15 mL Falcon tubes containing the saliva from jennies and foals were stored at –20 °C until the salivary cortisol analysis, which occurred between June 2018 and May 2019. For storage, the samples were thawed in the fridge and extracted from the gauze via centrifugation. The gauzes containing the samples were centrifuged for 15 min at 1000× *g*, and the extracted fluids were placed in 1.5 mL microtubes. These were then frozen again until analysis. The analysis was performed by trained professionals following EIA protocols, developed and validated by previously reported publications [48,49].

Additionally, *rapadura* was added to a standard curve, and no effect was observed in the performance of the assay.

### 2.7. Data Analysis

The data for the affiliative and inactivity behaviours were studied through the Poisson distribution, according to the PROC GLIMMIX of SAS, utilising a randomised block design with repeating measurements for the duration of occurrence of the observation in question, over time. Blocks were defined by the days of lactation. The model includes the effect of observation time in two different periods (before and after separation).

Data from the events of the behavioural categories agonistic, abnormal, eliminative and vocalisation were studied through the Poisson distribution, according to the PROC GLIMMIX of SAS, utilising a randomised block design with repeating measurements over time. Blocks were defined by days of lactation. The model includes the effect of observation time in two different periods (before and after separation).

For the salivary cortisol data, the Shapiro–Wilk test was conducted to analyse the normality of the residues, and the fixed effects were analysed by PROC GLIMMIX. The studied model included the effects of observation in two different periods (pre- and post-separation).

The milk yield data were analysed in a randomised block design. The statistical model considered the day of lactation to be a fixed factor and the animal (block) effect to be a random factor, defined by the RANDOM command. Fisher’s Least Significant Difference was used when the fixed factors were significant for both analyses. The PROC CORR procedure was used for determining the Pearson correlation between milk yield and salivary cortisol concentration for the jennies.

All analyses were done in the Statistical Analysis Software 9.4 (SAS) [50]; the adopted significance level was set at *p* <0.05.

## 3. Results

### 3.1. Behaviour

**Figure 1:**
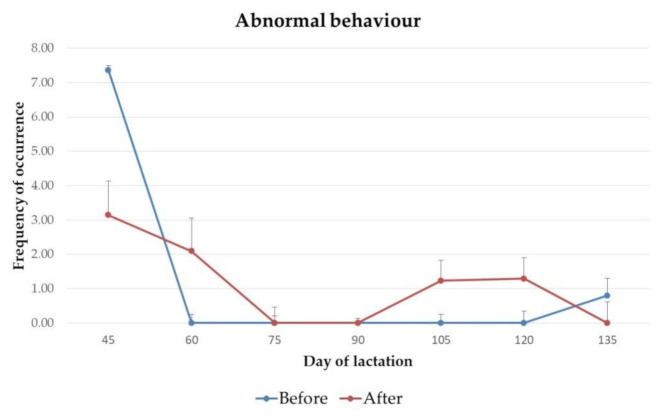
Mean frequency and standard deviation of occurrence of abnormal behaviour from jennies, before and after 2 h of separation from their foals for milking, on day 45 (*p* = 0.99), 60 (*p* = 0.99), 75 (*p* = 0.99), 90 (*p* = 1.00), 105 (*p* = 0.99), 120 (*p* = 0.99) and 135 (*p* = 1.00) of lactation.

**Figure 2:**
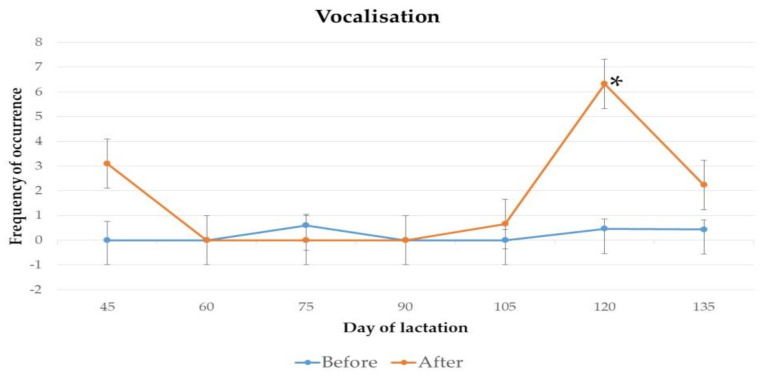
Mean frequency and standard deviation of occurrence of abnormal behaviour from jennies, before and after 2 h of separation from their foals for milking, on day 45 (*p* = 0.99), 60 (*p* = 0.99), 75 (*p* = 0.99), 90 (*p* = 1.00), 105 (*p* = 0.99), 120 (*p* = 0.03) and 135 (*p* = 0.09) of lactation. * indicates a statistically significant difference between frequencies.

**Figure 3:**
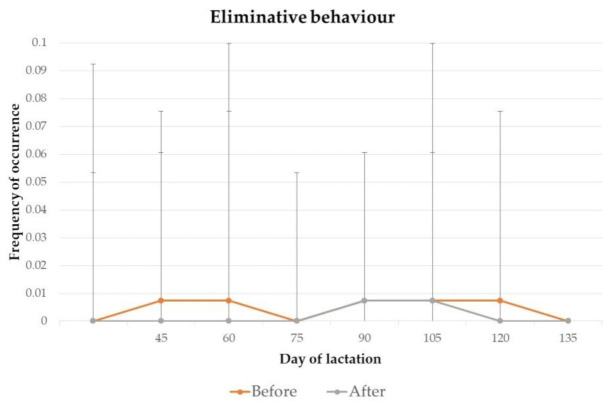
Mean frequency and standard deviation of occurrence of eliminative behaviour from jennies, before and after 2 h of separation from their foals for milking, on day 45 (*p* = 0.99), 60 (*p* = 0.99), 75 (*p* = 0.99), 90 (*p* = 0.99), 105 (*p* = 0.99), 120 (*p* = 0.99) and 135 (*p* = 0.99) of lactation.

**Figure 4:**
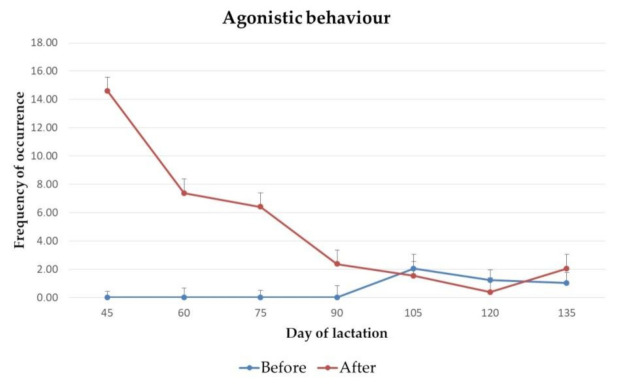
Mean frequency and standard deviation of occurrence of agonistic behaviour from jennies, before and after 2 h of separation from their foals for milking, on day 45 (*p* = 0.99), 60 (*p* = 0.99), 75 (*p* = 0.99), 90 (*p* = 0.99), 105 (*p* = 0.69), 120 (*p* = 0.40) and 135 (*p* = 0.40) of lactation.

The frequency of occurrence of abnormal behaviour, vocalisation, eliminative behaviour and agonistic behaviour of jennies on day 45, 60, 75, 90, 105, 120 and 135 of lactation, before and after 2 h of separation from their foals for manual milking, are presented in Figure 1, Figure 2, Figure 3 and Figure 4.

Statistically significant differences were found for the frequency of vocalisations (*p* = 0.03) from jennies, on day 120 of lactation. No significant differences were found for the frequency of abnormal, eliminative or agonistic behaviours throughout lactation.

**Figure 5:**
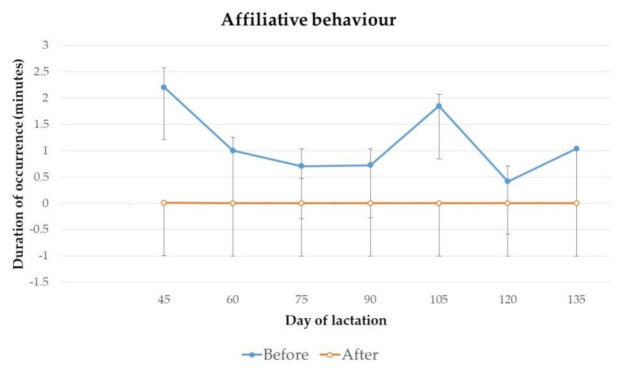
Mean duration and standard deviation of occurrence of affiliative behaviour from jennies, before and after 2 h of separation from their foals for milking, on day 45 (*p* = 0.99), 60 (*p* = 0.99), 75 (*p* = 0.99), 90 (*p* = 0.99), 105 (*p* = 0.99), 120 (*p* = 0.99) and 135 (*p* = 0.99) of lactation.

**Figure 6:**
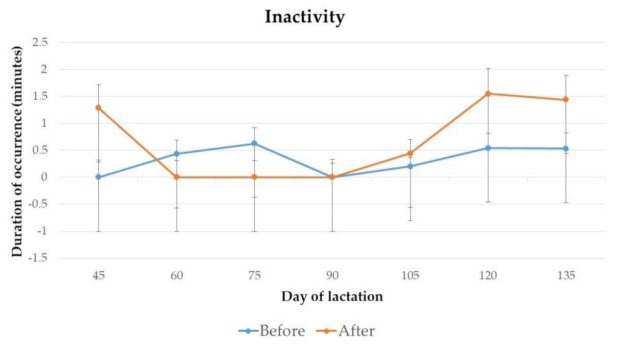
Mean duration and standard deviation of occurrence of inactivity from jennies, before and after 2 h of separation from their foals for milking, on day 45 (*p* = 0.99), 60 (*p* = 0.99), 75 (*p* = 0.99), 90 (*p* = 0.99), 105 (*p* = 0.44), 120 (*p* = 0.07) and 135 (*p* = 0.11) of lactation.

The duration of occurrence of affiliative behaviour and inactivity of jennies on day 45, 60, 75, 90, 105, 120 and 135 of lactation, before and after 2 h of separation from their foals for manual milking, are presented in Figure 5 and Figure 6.

No significant differences were found for the duration of affiliative behaviour and inactivity from jennies throughout lactation.

### 3.2. Salivary Cortisol Concentration

**Figure 7:**
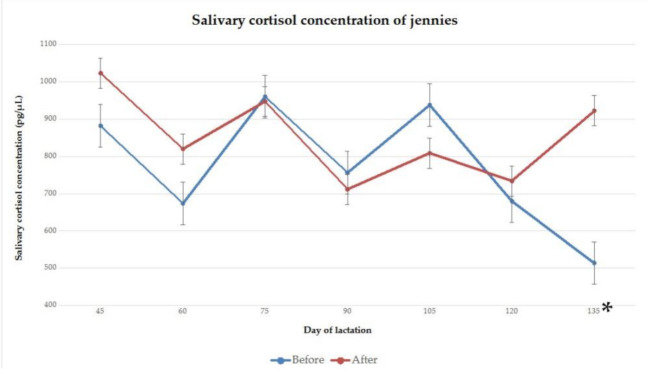
Mean and standard error of salivary cortisol concentration of jennies before and after 2 h of separation from their foals for milking, on day 45 (*p* = 0.41), 60 (*p* = 0.40), 75 (*p* = 0.94), 90 (*p* = 0.79), 105 (*p* = 0.44), 120 (*p* = 0.77) and 135 (*p* = 0.02) of lactation. * indicates a statistically significant difference.

The salivary cortisol concentration of jennies on day 45, 60, 75, 90, 105, 120 and 135 of lactation, before and after 2 h of separation from their foals for manual milking, are presented in Figure 7.

A statistically significant difference between salivary cortisol concentrations of jennies before and after separation was found on day 135 of lactation (*p* = 0.02), but not on day 45, 60, 75, 90, 105 or 120 of lactation (*p* > 0.05).

**Figure 8:**
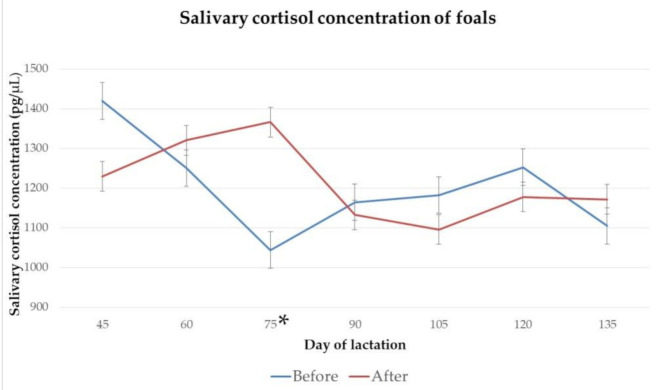
Mean and standard error of cortisol concentration of foals before and after 2 h of separation from their dams for milking, on day 45 (*p* = 0.19), 60 (*p* = 0.63), 75 (*p* = 0.03), 90 (*p* = 0.82), 105 (P = 0.62), 120 (*p* = 0.61) and 135 (*p* = 0.64) of lactation. * indicates a statistically significant difference.

The salivary cortisol concentrations of foals on day 45, 60, 75, 90, 105, 120 and 135 of lactation, before and after 2 h of separation from their dams for manual milking, are presented in Figure 8.

A statistically significant difference between salivary cortisol concentrations of foals before and after separation was found on day 75 of lactation (*p* = 0.03), but not on day 45, 60, 90, 105, 120 and 135 or lactation (*p* > 0.05).

### 3.3. Milk Yield

The average milk yield of the Pêga jennies was 566.4 ± 205.2 mL/animal/milking.

There was no correlation found between the milk yield and salivary cortisol concentration pre- or post-separation (Table 2).

## 4. Discussion

### 4.1. Behaviour

Behavioural observation is considered the most reliable and immediate way to assess the perception and interaction of an animal with its environment [51]. However, the social behaviour of donkeys has not been sufficiently studied [35].

In this study, foals were separated from the jennies for milking management starting at 45 days of age. It is likely that the age-dependent reduction in the proximity between jennies and their foals partially explains the small behavioural responses reported in this study [25].

Additionally, jennies and foals were allowed to remain in physical proximity and maintained vocal communication, as the jennies were aware of the location of their foals during the separation period. Donkey foals begin drinking water and graze by themselves at four weeks of age [26], and mule foals have been observed at distances of 50 to 100 m from their dams starting from the 3rd week of life, and distances of over 100 m after the 11th week [27].

The increase in the frequency of vocalisations post-separation on day 120 of lactation, in comparison to the pre-separation period, may have been generated by various factors. Vocalisations are important to maintain the interactions between jennies and their foals, e.g., to signal the start of nursing bouts or direct the activities of the foal [25], and the increase might represent the fact that the animals were not in visual contact. It is known that equines utilise vocal communication to express many emotional states, ranging from curiosity, playfulness, and anticipation to distress signals, discomfort, frustration, and stress [23]. As this increase was only observed on one assessment day, it is not possible to determine if the increased frequency of vocalisations were a response to potential stress from the 2-h separation period, or an attempt to communicate in the absence of visual contact. The relevance of vocalisations as indicators of emotionality in animals must be analysed together with the other parameters.

The absence of significant differences in behavioural measures may indicate that both jennies and foals coped with the 2-h separation period with biologically acceptable responses that maintained good levels of animal welfare. When faced with routine changes, external stressors, or poor welfare conditions, animals tend to demonstrate behavioural signs such as a rise in inactivity [25,26,27], elevated frequency of urination and defecation [43,44], altered social interactions [28] and a rise in abnormal behaviours [26].

Animals also tend to perform greater amounts of abnormal and agonistic behaviour when responding to adverse situations, which may relate to stressors caused by housing problems and/or improper handling [26]. Changes in the environment and activities performed by the animals may generate alterations in the social environment [45,46]. Such changes were not observed in the present study, in which management alterations and separation between dams and foals for 2 h did not significantly impact social behaviour. The study population of purebred Pêga donkeys is unique as they encounter a wealth of human–animal interactions throughout all developmental stages, which may have mitigated their responses.

It is important to mention that the absence of alterations in social behaviour of the jennies in this study may be explained by the fact their social groups were not changed, minimising potential conflicts related to hierarchy, and might also indicate that the 2-h separation from their foals did not challenge social stability.

Affiliative behaviours among equids provide several social benefits [47]. Some known affiliative behaviours described for equines are mutual grooming, touching between the muzzle and body, playing, approaching, and following [36,49], though the occurrence of grooming and greeting are considered rare in wild jennies [48]. The occurrence of these actions is influenced by age, reproductive stage, hormones, social structures, and ecological conditions [47,49,50], and their quality and quantity may also be altered according to the quality of their habitat. Animals may display an increased frequency of affiliative behaviours to ease tensions or in situations of low perceived risk; contrarily, they may decrease their frequency to avoid imminent conflicts or in risky situations [47].

The results presented here may indicate an absence of stress in jennies when separated from their foals for 2 h, but further investigations are needed in regard to the normal social behaviours of donkeys and how they vary in response to adverse situations.

Even though no significant behavioural alterations were observed in the post-separation periods, further investigations are required in respect to the affiliative, agonistic, abnormal, and eliminative behaviours and inactivity in order to determine if the 2-h separation is a stressor for these animals. The study is unique in that it monitored the responses of purebred Pêga jennies and their foals. The animals were handled on a routine basis for other purposes, and this could have mitigated the response to the separation.

### 4.2. Salivary Cortisol Concentration

Cortisol was measured from saliva. This collection method is non-invasive [51,52,53,54] and reflects the biologically active portion of the total circulating concentration [52,53,55]. It is thus less likely to induce increases in cortisol concentration when compared to plasma cortisol sampling [54,56,57]. The aversive stimuli of drawing blood in dairy jennies may cause more intense stress than milking [31]. Significant differences between pre- and post-separation samples were only observed from jennies on day 135 of lactation, and from foals on day 75 of lactation. On all other assessment days, no significant differences between pre- and post-separation samples were observed. The significant rise in cortisol concentration in jennies after separation on day 135 of lactation, when compared to before separation, might have been the result of changes in management, which caused some animals to remain separated for longer than 2 h.

Few studies regarding the response and adaptation of jennies to milking have been performed [13,31], and no significant variance has been found in salivary cortisol concentrations before and after milking, even though donkeys can show great reactivity to milking procedures [13].

The average concentration of salivary cortisol from jennies, before milking was 790 pg/μL (217.93 nmol/L), taken between 9:00 am and 10:00 am, and 840 pg/μL (231.72 nmol/L), taken between 12:00 pm and 2 pm, before and after separation, respectively. The sampling period can alter salivary cortisol concentrations, which can reach values of 531.72 nmol/L when taken after milking [13]. In non-pregnant mares, basal salivary cortisol concentrations vary between 110.34 nmol/L and 331.03 nmol/L [58]. Significant differences have been reported between salivary cortisol concentrations of donkey stallions and equine mares or geldings [59,60], which have been ascribed to species variation [56].

The time of day in which samples are taken also affects results, due to circadian rhythms. Cortisol concentrations follow a clear diurnal pattern in horses, with the highest concentrations in the morning and the lowest in the late afternoon and evening [58,61,62,63]. This trend has also been observed in donkeys, with high plasma cortisol levels found in jennies milked at 8:00 am and lower values in groups milked at 4 pm [31].

As salivary cortisol levels were not measured throughout the day without routine changes, in the present study, and the circadian rhythm may influence basal cortisol levels, it is uncertain whether the absence of significant difference between cortisol levels before and after separation and milking is due to the absence of stress for the animals or lower basal levels at later times of the day [31].

It has been stated that inherent diurnal rhythms can be easily disturbed by minor challenges [59,61] and factors such as weather and ambient temperature, and interactions within groups may cause transient alterations [59]. Experimentally induced increases in salivary cortisol are often relatively small, and hardly exceed the range of physiological variations [54]. More research is needed regarding the variations in cortisol level in jennies according time of day and seasons of the year [13,31,56].

In horses, stressful events like separation from conspecifics acutely stimulates cortisol release [62,64], but they quickly habituate to these situations, and there are no lasting effects on diurnal rhythm. Furthermore, repeated stressful events also result in subsequent decreased cortisol levels [60].

The average concentration of salivary cortisol from foals before the separation from their dams was numerically higher than those of jennies, in agreement with studies performed in horses, which reported higher cortisol levels in suckling foals compared to their dams [59]. The elevated levels of cortisol in foals may be due to immaturity, following the same pattern found in gilts and humans [65,66,67], in which cortisol levels are initially high and gradually lower while forming a circadian rhythm.

The fact that the 2-h separation period, in all but two assessments, did not generate a significant difference between pre- and post-separation salivary cortisol concentrations in jennies or foals may indicate that this interval was not a stressful factor capable of altering the HPA axis, and may not compromise the welfare of the animals involved. Therefore, the ability of jennies and foals to adapt over time in response to stress remains unclear.

### 4.3. Milk Yield

The milk yield data differ from other studies, which, working with jennies of the Pêga breed in an extensive farming system in the drought season and without nutritional supplementation, reported an average milk yield in two daily milkings of 0.614 kg/day [68].

Ragusana donkeys receiving hay ad libitum and 3.5 kg feed/day have higher yields than those reported in this study, ranging from 0.56 to 0.59 kg/milking, from two and eight milkings, respectively [18]. This suggests that the difference in milk production of the different breeds could be linked to their diet [69].

The ejection of alveolar milk may be altered by stress, due to oxytocin concentration changes and myoepithelial contraction [42]. Jennies submitted to milking without previous training show lower milk yield when compared to jennies previously habituated to milking management, possibly due to reduced oxytocin supply via vasoconstriction or blocking of its receptors in the myoepithelial cells of the udder alveoli [13]. If we consider the density of Pêga asinine milk to be 1.03 g/mL [69], the milk yield was 0.6 kg/animal/milking, which represents approximately 0.25% of the jennies’ average body weight. Since these animals may be milked two [10] to eight times a day [18], the total milk yield from these jennies may be up to 4.8 kg/animal/day.

Milk yield remained constant for all animals after the 2-h separation period on all days of lactation, and there was no correlation between the volume of milk produced and cortisol concentration pre- and post-separation, thus separation was not a stressor that impaired milk ejection. However, many other factors can alter milk ejection, and must be considered before concluding that there was no stressor present during the 2 h separation.

It is important to consider the fact that the animals had interactions with humans on a regular basis and some of the management practices involved short-term separation of jennies and foals.

## 5. Conclusions

The results presented in this study show that the behavioural categories assessed for the jennies were only mildly altered by the 2-h separation from their foals. The only behavioural variable that showed significant changes was vocalisation frequency, which may express social signalling in the absence of visual contact, and this was only observed on one assessment day.

The 2-h separation period also failed to generate significant changes in the majority of salivary cortisol concentration levels of jennies or foals, with the exception of one assessment in each animal category. Therefore, it does not appear to be a stressor capable of altering the HPA axis. Additionally, there was no apparent relationship between milk ejection and salivary cortisol concentrations.

We acknowledge several limitations to this study, such as the absence of a control group, the lack of true measures of basal salivary cortisol, and the low quantity of milk yield measurements to support more robust conclusions. Behavioural observations of foals will enhance our understanding of the impact of separation on their welfare. Further research is needed to determine whether the separation of these animals is indeed a stressor that could result in severe welfare problems. Additional studies are also required to determine the long-term consequences of the separation event, as well as the results of more frequent separation periods, on the lifelong trajectory of these animals.

Considering these results, it is important to emphasise that they are limited to one group of purebred Pêga jennies and foals in Brazil. It is possible that their responses were confounded by the fact that these animals were handled non-aversively on a regular basis. We hope that this study is useful to people interested in milking donkeys.

## Figures and Tables

**Table 1:** Experimental ethogram utilised to assess jennies’ behaviour.

**Affiliative**	
Mutual grooming	Behaviour in which two donkeys use their teeth to simultaneously nibble any of each other’s body parts.
Licking	Licking any part of the body of another donkey.
Body sniffing	Sniffing the neck, withers, flank or tail of another donkey which may or may not reciprocate.
Approaching	Moving to within 1 m of another donkey that does not immediately move away and staying there for at least 10 s without initiating physical contact with it.
Touching	Touching another donkey at the neck or head, which may or may not reciprocate.
**Agonistic**	
Kicking	Rapid lifting of one or both hind limbs off the ground, directed towards another donkey or the observer, in an attempt to hit them, with the ears laid back.
Pushing	Pressing head, neck, chest or shoulder against another donkey, making them move away.
Chasing	Rapid movement toward another donkey and pursuit for a distance of over three body lengths, with the ears laid back, head raised and mouth closed.
Biting	Extension of head and neck towards another donkey, with the ears laid back, head raised and mouth open, closing teeth on its body.
Fighting	Pursuing another donkey for a distance of over three body lengths, with ears laid back, head raised and mouth open, attempting to close teeth on its body.
**Abnormal**	
Biting the stalls or structures	Grasping of structures with incisive teeth, which may be followed by simultaneous arching of the neck and sucking of air (cribbing).
False licking	Behaviour in which the animal slowly places its tongue on the borders of the stall or trough while keeping it still and stiff, so the action does not represent true licking.
Pawing	Vigorous and persistent stomping of limbs on the ground.
**Eliminative**	
Urinating	Elimination of urine.
Defecating	Elimination of faeces.
**Vocalisations**	
Vocalisations	Expression of vocal communication, such as whinnies, snores, snorts, groans or screams.
**Inactivity**	
Inactivity	Absence of movement or other actions.

**Table 2:** Correlation between milk yield and salivary cortisol concentrations of jennies pre- and post-2 h of separation from their foals.

	Milk Yield (mL/day)	Cortisol before 2 h Separation (nmol/L)	Cortisol after 2 h Separation (nmol/pL)
Milk yield (mL/day)	1.00	−0.131	−0.044
Cortisol before 2 h separation (nmol/L)		1.00	0.432
Cortisol after 2 h separation (nmol/pL)			1.00

## Data Availability

Publicly available datasets were analyzed in this study. This data can be found here: https://data.mendeley.com/datasets/7n9t5fn99b/2.
